# Test-Retest Reliability, Validity, and Minimal Detectable Change of the Measurement of Lower Limb Muscular Strength with Handheld Dynamometry in Patients Undergoing Hemodialysis

**DOI:** 10.1155/2022/5330608

**Published:** 2022-06-15

**Authors:** Borja Perez-Dominguez, Aida Lopez-Brull, Maria Plaza-Carrasco, Jose Casaña-Granell, Alicia Garcia-Testal, Josep Benitez-Martinez

**Affiliations:** ^1^Department of Physiotherapy, University of Valencia, Valencia, Spain; ^2^Hospital de Manises, Valencia, Spain

## Abstract

**Background:**

Chronic kidney disease is an exponentially growing medical and economic worldwide problem. There are specific elements used to assess patient's functional capacity loss and overall deterioration in order to determine the patient's clinical status, and muscle impairment is one of the most common. It is therefore necessary to develop reliable and applicable methods to determine muscle impairment in patients with chronic kidney disease

**Methods:**

This is a prospective, nonexperimental, descriptive methodological investigation performed in patients undergoing hemodialysis. This study analyzes the reliability and validity of muscle strength assessments performed with handheld dynamometry in patients with chronic kidney disease undergoing hemodialysis.

**Results:**

Results show overall high reliability and validity in the assessment of muscle strength of the lower limbs

**Conclusion:**

To our knowledge, this is the first study to assess handheld dynamometry in patients undergoing hemodialysis, presenting promising results with a relatively affordable and easily applicable method.

## 1. Introduction

Chronic kidney disease (CKD) is an exponentially growing health and social problem where many comorbidities and clinical complications can be found [1]. Patients that suffer from CKD are often sedentary and have high mortality risk factors [2]. They are defined, among by other characteristics, with a deterioration of their physical condition and health-related quality of life. In late stages of CKD, these clinical situations imply muscle weakness and functional impairment, along with other problems such as muscle atrophy or depression [2–5].

Therefore, muscle strength is negatively affected by the disease, and it also serves as a powerful indicator of the nutritional and clinical state of the patient that suffering from CKD [6]. “Sarcopenia” is a term which refers to the loss of muscle tissue and function related to aging and chronic diseases, and due to the catabolic environment generated in CKD with altered uremic states, it is rapidly developed due to an imbalance between muscle regeneration and degeneration [7].

Weakness, fatigue, and muscle cramps could appear in 6–11% of the hemodialysis sessions [8], and they are considered substantial factors that affect the patient's functional capacity [9, 10]. Amongst morphological alterations found in patients with CKD, it is common to observe a decrease in muscular cross section size [11, 12] that affects majorly anaerobic type II fibers.

There is a recently defined term for patients that suffer from CKD called “muscle wasting” that refers to nutritional and catabolic alterations which occur due to alterations in protein synthesis, sedentarism, and catabolic states that are associated with the disease [13]. In its most severe cases, the energetic protein wasting occurring in renal disease is defined as “cachexia,” and these states are usually associated with muscular weakness.

Because there is a clear relationship between muscle impairment and the clinical deterioration of the patient, there is a rising awareness of the importance to develop muscle strength assessment methods that are reliable and valid. Therefore, the objective of this study is to evaluate the reliability, validity, and minimal detectable change of the assessment of muscular strength using a handheld dynamometer.

## 2. Materials and Methods

### 2.1. Design

This study was a prospective, nonexperimental, descriptive methodological investigation performed in patients with CKD undergoing hemodialysis. Reliability and validity were assessed in two identical sessions with a one-week interval between them by comparing the results obtained in both sessions. The researcher in charge of the assessments was a physical therapist specialized in sports and strength conditioning that had been previously trained in using handheld dynamometry. The researcher in charge of the assessments could not be blinded to the prior results in the first session, but to assure blinding, participants were not told their first session results. This study was conducted in a hospital's hemodialysis unit, between March 9, 2016, and March 16, 2016.

### 2.2. Participants

Patients that suffered from end-stage CKD who underwent hemodialysis were enrolled. All participants were enrolled and screened for study eligibility in a consecutive sample when the main researcher was present in the hemodialysis unit. Every participant was assessed for eligibility by their medical record and were granted permission by the head nephrologist of the hemodialysis unit. All participants were able to walk without aid. Every participant was given verbal and written information regarding the procedure and objective of the study and were also asked to give written consent to enroll if they were willing to participate. All the participants were informed, and it was made clear that their participation was voluntary and withdrawal could be done at any time.

Inclusion criteria were for the participant to have been treated with hemodialysis for at least 3 months and having a stable medical condition to participate in physical activity. Exclusion criteria included (1) myocardial infarction 6 weeks prior to the intervention, (2) unstable cardiovascular disease that might worsen with exercise, (3) above-knee lower limb amputation, (4) ischemic brain disease, (5) musculoskeletal or respiratory condition that could worsen with exercise, or (6) inability to perform functional tests for several reasons, such as language barrier.

### 2.3. Assessments

**Figure 1:**
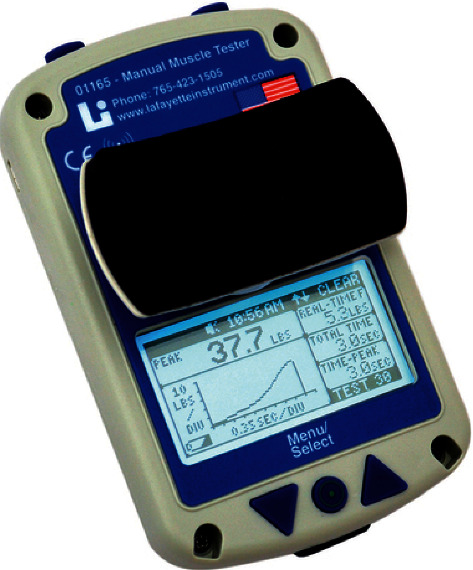
Nicholas manual muscle tester.

A handheld dynamometer (Nicholas manual muscle tester, Lafayette) shown in Figure 1 was used following an adaptation of a standardized protocol created by the manufacturer. The rationale behind this decision was that there are no previously developed muscle assessment protocols for patients undergoing hemodialysis, so creating an adapted version of the standard protocol provided by the manufacturer seemed the more applicable option. The assessment protocol was adapted to the participant's position, either sitting or lying, as patients on hemodialysis usually are positioned during the session.

The assessment took place during the first 2 hours of the hemodialysis session to avoid possible complications, such as muscular fatigue or hypotension. The researcher in charge of the assessments, a trained physical therapist specialized in therapeutic exercise, was positioned in mechanical advantage for every measurement, parallel to the axis on which the movement was going to take place and placed the dynamometer in the same anatomical point as described in the protocol. The participant was also placed in a comfortable position, also described in the adapted protocol, to facilitate the muscular contraction.

Participants were firstly explained the movement they had to do, and prior to the assessment, they were given the chance to perform a “trial” contraction. Once the researcher assured the movement was made clear, participants were asked to perform a maximal isometric contraction, sustained for 3 seconds, and the force exerted was registered in Newton (N). Assessments were performed for both lower limbs and for every muscular group.

#### 2.3.1. Muscle Group Assessment Protocol

The following muscle groups were assessed for both lower limbs: quadriceps, iliopsoas, triceps surae, hip adductors, hip abductors, and hamstrings.

*(1) Quadriceps*. The participant was placed in either sitting or lying position with a ball beneath the assessed knee, creating a 45° flexed angle. The researcher was positioned in mechanical advantage and placed the dynamometer on the anterior side of the distal third of the participant's fibula. The participant was asked to deliver a knee extension maximal isometric contraction for 3 seconds.

*(2) Hip Flexors*. The participant was placed with a 45° flexed knee and hip. The researcher was positioned in mechanical advantage and placed the dynamometer in the distal third of the participants femur. The participant was asked to do a hip flexion maximal isometric contraction for 3 seconds.

*(3) Triceps Surae*. The participant's knee was completely extended, and a ball was placed beneath the distal third of the fibula. The researcher was positioned in mechanical advantage, and the dynamometer was placed in the plantar side of the participant's forefoot. The participant was asked to deliver a plantar flexion maximal isometric contraction for 3 seconds.

*(4) Hip Adductors*. The participant had the knee fully extended. The researcher was positioned in the opposite side to the assessed limb in mechanical advantage, and the dynamometer was placed on the internal border of the distal third of the femur. The participant was asked to deliver a hip adduction maximal isometric contraction.

*(5) Hip Abductors*. The participant had the knee fully extended. The researcher was positioned in the same side to the assessed limb in mechanical advantage, and the dynamometer was placed on the external border of the distal third of the femur. The participant was asked to deliver a hip abduction maximal isometric contraction.

*(6) Hamstrings*. The participant was placed with a 45° flexed knee and hip. The researcher was positioned in front of the participant in mechanical advantage, and the dynamometer was placed in the posterior side of the participant's distal third of the femur. The participant was asked to deliver a knee flexion maximal isometric contraction.

### 2.4. Statistical Analysis

For this study, two measurements were performed in two different times weekly apart for every participant and for every muscle group. Both measurements were performed by the same researcher. The SPSS package version 26 for iOS was used for data management and analysis, and the level of significance was predetermined at *p* < 0.05 for all analyses. Indeterminate results were false-positive and were incorporated in the final analysis.

To address intraobserver reliability, because this study dealt with a quantitative variable (muscle strength), the intraclass correlation coefficient (ICC) [14] was used (model alpha, 2-way random effects model). Intraobserver point estimates of the correlation and ICC values were based on those provided by Portney and Watkins [15] interpreted as excellent (0.90), good (0.75–0.89), moderate (0.50–0.74), or poor (<0.50).

In order to understand the extent to which the test really assesses muscular strength, concurrent validity was analyzed. To do so, Pearson's product moment correlation coefficient (*r*) was used to analyze the correlation between the test and retest scores for every muscular group. Pearson's correlation index establishes linear correlation with a value that ranges from −1 to 1, and the closer the result is towards either value, the higher the correlation is.

Minimal detectable change (MDC) establishes the minimum change in a measurement necessary to conclude that the difference is not attributable to error. MDC in this study was calculated using formulas presented in previous studies [16, 17].

## 3. Results

**Figure 2:**
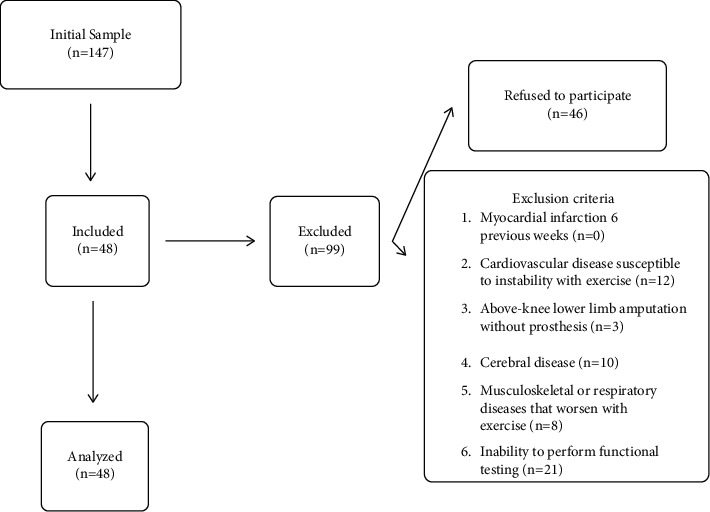
Participant flow chart.

Figure 2 shows the flow of participants throughout the study. Out of 147 initially screened participants, 48 were enrolled in the study. Every participant completed the assessments. A summary of their baseline demographic and clinical data is presented in Table 1. The participants had a mean (SD) age of 71.8 (15.6) years, and 33 of them were males and 15 were females. Strength values are presented in Newton (N).

### 3.1. Reliability Results

Results are collected in Table 2 and show that there are good (0.75–0.89) reliability values when assessing muscle strength with a handheld dynamometer in the following muscular groups: quadriceps (ICC = 0.81 for the right lower limb and ICC = 0.84 for the left), hip flexors (ICC = 0.82 for the right lower limb and ICC = 0.80 for the left), right hip adductors (ICC = 0.82), and hamstrings (ICC = 0.91 for the right lower limb and ICC = 0.82 for the left). Reliability is moderate (0.50–0.74) regarding the right triceps surae (ICC = 0.76), hip abductors (ICC = 0.70 for the right lower limb and 0.76 for the left), and left hip adductors (ICC = 0.79). Poor (<0.50) reliability was only found when assessing the left triceps surae (ICC = 0.38).

### 3.2. Validity Results

Validity analyses are shown in Table 3. Results show different levels of correlation among the assessment of the muscular groups. There are high correlation levels, which authors consider to be above 0.80, in the assessment of quadriceps (0.83 for the right and 0.86 for the left), hip flexors (0.82 for the right and 0.80 for the left), right hip abductors (0.84), and hamstrings (0.92 for the right and 0.85 for the left).

Mild correlation levels, considered in a range between 0.6 and 0.8, were shown in the right triceps surae (0.76), hip adductors (0.70 for the right and 0.77 for the left), and the left hip abductor (0.79). Low correlation levels, understood to be below 0.60, were only shown in the left triceps surae (0.39).

### 3.3. Minimal Detectable Change

Results are shown in Table 4. The minimal difference in order to appreciate detectable changes, or the MDC, was established for the quadriceps (33.6 N for the right lower limb and 27.9 N for the left), hip flexors (29.3 N for the right lower limb and 30.4 N for the left), triceps surae (31.6 N for the right lower limb and 48.7 N for the left), hip adductors (23.2 N for the right lower limb and 23.6 N for the left), hip abductors (20.6 N for the right lower limbs and 28.7 N for the left), and hamstrings (17.9 N for the right lower limb and 23.6 N for the left).

## 4. Discussion

This study assessed reliability, validity, and minimal detectable change for the main muscular groups of the lower limbs, showing high overall reliability and validity results. To our knowledge, this is the first study to assess lower limb muscular strength in patients undergoing HD with a handheld dynamometer. Handheld dynamometry presents several advantages respective to other assessment instruments, involving its relative low cost or the possibility to apply during the dialysis session requiring no extra time for the patients. Other studies assessed muscular strength with a handheld dynamometer in different populations, such as hematologic pathologies [18], patients with knee osteoarthritis [19], or patients about to receive a total knee arthroplasty [20]. Muscular strength has also been assessed in patients undergoing HD, but with different, more expensive methods, like the response seated leg curl thigh extension system [21], a digital isometric dynamometer [22], a dynamometric system PC-2 SDT [23], or specific equipment to assess physical condition [24].

Reliability was analyzed using the ICC, and most of the assessments showed high values (>0.80) [24], so we can consider the use of a handheld dynamometer as a reliable instrument to assess muscular strength in patients undergoing hemodialysis. Only one of the assessments showed low reliability (left triceps surae, with an ICC = 0.38). This could be explained because of how big the area is where the dynamometer was placed, being much more comfortable for the hip flexors (distal third of the femur) than triceps surae (plantar side of the forefoot).

One of the major limitations in this study could be that assessments were taken during the dialysis session, so absolute values might not reflect completely the strength the muscle is able to exert. Future studies should assess if reliability values are also high when assessing muscular strength before the dialysis session. More so, there is no assessment protocol developed for patients under HD, and many of the existing protocols [25] cannot be applied to a dialyzed patient because the patient cannot be placed in a lateral or prone position.

Validity also showed relatively high results, but these must be carefully considered because the interpretation of validity depends on the context and purposes of the study [26]. Our study, as mentioned in reliability results, also found that handheld dynamometry was a valid muscle strength assessment in all muscle groups, but the left triceps surae showed a Pearson's correlation index of 0.39. As mentioned before, these inconsistencies could be due to the protocol used, so this must be taken under consideration for future studies.

Finally, regarding the MDC, there are previous studies that also assess it in lower limb strength, but in healthy individuals [27], the comparison with our results must be cautious. Our results are consistent with those found on this study, and this similarity could be related to both study samples showing low comorbidity values. Interestingly, Mentiplay et al. also found high variability in the results of muscular strength of the triceps surae, so these results should be further analyzed by future studies.

## Figures and Tables

**Table 1:** Demographic characteristics of the participants.

Age, median (SD) (years)	71.8 (15.6)

Gender, *n* (%)	
Male	33 (69)
Female	15 (31)
Weight, median (SD) (kg)	74.8 (14.7)
Height, median (SD) (cm)	164.1 (10.2)
Body mass index, median (SD) (kg/m^2^)	27.9 (5.4)
Albumin, median (SD) (mg/dL)	3.9 (0.3)
Creatine, median (SD) (mg/dL)	7.1 (1.9)
Glycolyzed haemoglobin, median (SD) (g/dL)	5.8 (0.9)

CKD diagnosis	
Diabetes mellitus	4
Glomerular nephritis	7
Lupus	1
Pyelonephritis	2
Polycystosis	1
Others	30
Hypertension	3

Diabetes	
No	29
Diabetes type I	3
Diabetes type II	16

Smoker	
No	37
Yes	11

Dialyzer	
FX100	9
FX80	24
FX60 Classix	13
F70S	1
FX10	1

Charlson's index score	
2	8
3	2
4	2
5	4
6	6
7	12
8	6
9	4
10	3
11	1

**Table 2:** Test-retest: reliability outcomes.

Muscle group	Test: mean (SD) median (min-max)	Retest: mean (SD) median (min-max)	ICC **(95% CI)**	*p* value
Quadriceps R (Newton)	84.6 (33.4) 79.2 (32.1–189.2)	79.0 (29.7) 76.3 (32.2–175.2)	0.81 (0.69–0.89)	0.186
Quadriceps L (Newton)	84.5 (26.9) 84.4 (27.3–154.7)	77.9 (29.4) 76.4 (27.1–159.1)	0.84 (0.69–0.91)	*p* < 0.05
Hip flexors R (Newtons)	78.8 (29.6) 82.8 (12.4–144.7)	73.5 (28.7) 70.9(13.3–150.6)	0.82 (0.69–0.90)	0.244
Hip flexors L (Newtons)	77.3 (28.1) 78.7 (19.9–144.5)	72.0 (29.2) 66.1 (23.5–162.8)	0.80 (0.67–0.89)	*p* < 0.05
Triceps surae R (Newtons)	60.7 (27.3) 55.5 (16.6–154.4)	58.4 (27.4) 53.5 (16.8–155.6)	0.76 (0.60–0.86)	0.489
Triceps surae L (Newtons)	63.5 (26.4) 59.0 (22.5–154.1)	56.4 (25.3) 54.6 (0.0–140.2)	0.38 (0.11–0.59)	0.168
Hip adductors R (Newtons)	57.9 (23.2) 54.6 (23.2–123.6)	56.0 (20.3) 56.6 (22.2–125.6)	0.82 (0.70–0.89)	0.739
Hip Adductors L (Newtons)	59.6 (22.2) 58.2 (22.4–129.5)	58.4 (21.8) 54.7 (25.1–117.0)	0.79 (0.66–0.88)	0.573
Hip abductors R (Newtons)	61.0 (15.4) 59.8 (28.2–111.0)	60.7 (16.1) 59.6. (35.4–112.0)	0.70 (0.52–0.82)	0.638
Hip Abductors L (Newtons)	63.7 (21.2) 61.8 (29.5–126.0)	65.4 (25.2) 59.2 (26.8–161.7)	0.76 (0.61–0.86)	0.491
Hamstrings R (Newtons)	75.0 (25.0) 73.5 (12.1–133.4)	70.5 (24.4) 65.9 (22.8–130.7)	0.91 (0.81–0.95)	*p* < 0.05
Hamstrings L (Newtons)	72.8 (24.6) 74.8 (25.3–123.6)	65.5 (23.6) 62.9 (22.6–125.5)	0.82 (0.59–0.90)	*p* < 0.05

ICC: intraclass correlation coefficient—confidence intervals are also presented up to 95% of the mean; MDC: minimal detectable change, R: right; L: left; SD: standard deviation.

**Table 3:** Test-retest: validity outcomes.

Muscle group	Pearson's correlation index	*p* value
Quadriceps R	0.83	*p* < 0.05
Quadriceps L	0.86	*p* < 0.05
Hip flexors R	0.83	*p* < 0.05
Hip flexors L	0.81	*p* < 0.05
Triceps surae R	0.76	*p* < 0.05
Triceps surae L	0.39	*p* < 0.05
Hip adductors R	0.70	*p* < 0.05
Hip Adductors L	0.77	*p* < 0.05
Hip abductors R	0.84	*p* < 0.05
Hip Abductors L	0.79	*p* < 0.05
Hamstrings R	0.92	*p* < 0.05
Hamstrings L	0.85	*p* < 0.05

R: right; L: left.

**Table 4:** Minimal detectable change outcomes.

Muscle group	Minimal detectable change (min-max)	*p* value
Quadriceps R (Newton)	33.6 (25.6–43.6)	*p* < 0.05
Quadriceps L (Newton)	27.9 (20.5–38.0)	*p* < 0.05
Hip flexors R (Newton)	29.3 (22.2–38.2)	*p* < 0.05
Hip flexors L (Newton)	30.4 (23.1–39.3)	*p* < 0.05
Triceps surae R (Newton)	31.6 (24.3–40.3)	*p* < 0.05
Triceps surae L (Newton)	48.7 (39.4–58.1)	*p* < 0.05
Hip adductors R (Newton)	23.2 (17.7–29.9)	*p* < 0.05
Hip Adductors L (Newton)	23.6 (18.0–30.3)	*p* < 0.05
Hip abductors R (Newton)	20.6 (15.9–26.0)	*p* < 0.05
Hip Abductors L (Newton)	28.7 (22.1–36.7)	*p* < 0.05
Hamstrings R (Newton)	17.9 (13.0–23.3)	*p* < 0.05
Hamstrings L (Newton)	23.6 (16.6–35.0)	*p* < 0.05

R: right; L: left.

## Data Availability

The statistical data used to support the findings of this study are included within the article.

## Additional Points

Due to the nature of this study, trial prospective registration was not performed. The assessments done for this analysis belonged to a series of trials performed in the same sample.
